# Identification of antifungal principle in the solvent extract of an endophytic fungus *Chaetomium globosum* from *Withania somnifera*

**DOI:** 10.1186/2193-1801-2-37

**Published:** 2013-02-06

**Authors:** Susheel Kumar, Nutan Kaushik, Peter Proksch

**Affiliations:** 1 TERI University, 10 Institutional Area, Vasant Kunj, New Delhi, 110 070 India; 2 Habitat Center, The Energy and Resources Institute (TERI), Lodhi Road, New Delhi, 110003 India; 3 Institut für Pharmazeutische Biologie und Biotechnologie, Heinrich-Heine-Universität Düsseldorf, Düsseldorf, 40225 Germany

**Keywords:** *Chaetomium globosum*, Endophyte, *Withania somnifera*, Antifungal, *Sclerotinia*

## Abstract

Extracts of *Chaetomium globosum* EF18, isolated as endophytic fungus from *Withania somnifera*, were found effective against *Sclerotinia sclerotiorum.* Ethyl acetate and methanol extracts were more effective than hexane extract showing >80% growth inhibition. Bioactive compound (antibiotic Sch 210971, m/z 445 and λ_max_ 290) having antifungal activity against *S. sclerotiorum* has been isolated in pure form from the ethyl acetate extract following bioassay guided fractionation. Apart from this compound other fractions of polar to medium polarity were also found effective. Fraction no. VIII from VLC (Vacuum liquid chromatography) column of ethyl acetate extract was most active having IC_50_ value 35.4 μg/ml.

## Introduction

The need for new and useful compounds to provide protection and relief to crop plants from pests and thereby sustenance of food production for human consumption is ever growing. Plant diseases have been causing devastating effects on crop plants and human life since the human civilization evolved (Agrios 2005). To combat such diseases, safer and greener chemicals have to be developed because conventional chemicals are posing greater threat to ecology and biodiversity and also causing ill effects to human health. Microbes and their compounds are emerging as alternative strategies for pest control (Montesinos 2003).

Many endophytic fungi and their metabolites have been reported to have insecticidal and fungicidal activity (Kumar et al. 2008). In our effort to isolate bioactive endophytic fungi, we isolated various endophytic fungi from *Withania somnifera*, among which *Chaetomium globosum* EF18 showed good activity against *Sclerotinia sclerotiorum, Fusarium oxysporum* and *Rhizoctonia solani* (Kumar et al., communicated paper). *S. sclerotiorum* is non-specific and omnivorous pathogen causing diseases to plants belonging to 75 families, 278 genera, and 408 species (Boland and Hall 1994). Some of the major crops affected by *S. sclerotiorum* are brassicas, potato, chickpea, pea, sunflower, beans, carrot, lettuce, soybean, kiwifruit and grapes. Although the most common disease caused by *S. sclerotiorum* is white mould, yet cottony rot, watery soft rot, stem rot, drop, crown rot, blossom blight are also of common occurrence in crop plants causing yield loss up to 100% (Purdy 1979). 1.5 million Tones of yield reduction due to *Sclerotinia* infection in soybean have been reported in US during 2004 (Wrather and Koenning 2006). This paper describes the isolation of active metabolites of endophytic fungus through bioassay mediated fractionation using VLC, Column chromatography and preparative HPLC. Bioactivity was tested against *S. sclerotiorum* during the experimentation.

## Results

**Figure 1:**
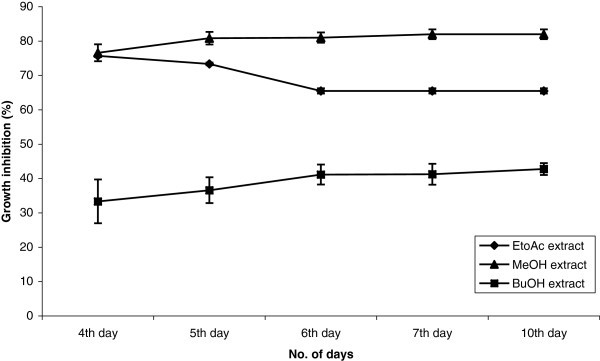
**Effect of different extracts of*Chaetomium globosum*isolate EF18 against plant pathogenic fungi,*Sclerotinia sclerotiorum*(CD0.05 = 6.864, CD0.01 = 9.261).**

Ethyl acetate, methanol (a portion of EtoAc extract was partitioned between methanol and hexane and then assayed to see if polarity has major effect on antifungal activity) and butanol extracts of endophytic fungus *C. globosum* were tested at 250 μg/ml and 500 μg/ml against *S. sclerotiorum*. Methanol extract showed 76.58% mycelial growth inhibition (GI) at 500 μg/ml on 4^th^ day, which further increased to 80.83% on 5^th^ day. Ethyl acetate extract was effective at 500 μg/ml having 75.68% growth inhibition on 4^th^ day. No significant difference was observed in the growth inhibition caused by ethyl acetate extract and methanol extract on 4^th^ day. Butanol extract was least effective showing 33.32% growth inhibition at 500 μg/ml on 4^th^ day. Graphical representation of GI over no. of days is given in Figure 1.

**Figure 2:**
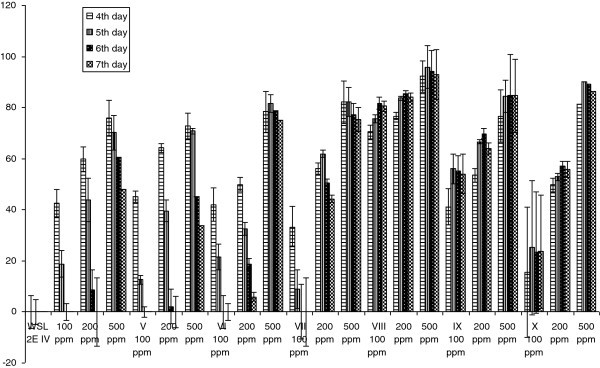
**Graphical representations of effect different concentration of VLC fractions of*C. globosum*EF18 on radial growth of*S. sclerotiorum*(values on Y axis are % growth inhibition).**

Ethyl acetate extract was further fractionated by VLC on silica gel. On the basis of HPLC and LC-MS analysis it was observed that all the VLC fractions were mixtures of many compounds. When tested for their bioactivity, fraction WSL-2E_VIII exhibited maximum mycelial growth inhibition of 95.9%, 83.7% and 75.6% at 500 μg/ml, 200 μg/ml and 100 μg/ml respectively (Table 1). Fraction WSL-2E_VI, WSL-2E_VII, WSL-2E_IX and WSL-2E_X were statistically at par and caused more than 80% growth inhibition at 500 μg/ml. Fraction IV and V exhibited least growth inhibition, ~70% at 500 μg/ml, among all the fractions. Figure 2 shows growth inhibition caused by these fractions at different concentrations. IC_50_ values were calculated for these fractions and are provided in Table 2, which shows that lowest IC_50_ of 40.57 μg/ml and 35.4 μg/ml on 4^th^ and 5^th^ day respectively was observed in fraction no. VIII. Chi-square values and regression equation have also been provided in the Table 2. Fraction no. IX stands next to fraction VIII showing IC_50_ value of 80.14 μg/ml on 5^th^ day of observation.

**Table 1:** **Effect of different fractions obtained from vacuum liquid chromatography of ethyl acetate extract of*Chaetomium globosum*EF18 on growth of*Sclerotinia sclerotiorum***

Sl no.	Fractions	Growth inhibition (%) on 5^th^ day		
		100 μg/ml	200 μg/ml	500 μg/ml
1	WSL 2E IV	18.8 ± 4.8	43.9 ± 5.4	70.2 ± 8.5
2	WSL 2E V	12.6 ± 6.8	39.6 ± 1.7	70.8 ± 4.4
3	WSL 2E VI	21.6 ± 1.0	32.5 ± 5.1	81.6 ± 2.4
4	WSL 2E VII	9.0 ± 3.4	61.9 ± 7.5	82.4 ± 1.4
5	WSL 2E VIII	75.6 ± 5.7	83.7 ± 1.6	95.9 ± 0.8
6	WSL 2E IX	56.0 ± 8.5	66.7 ± 5.7	84.5 ± 0.8
7	WSL 2E X	25.2 ± 6.4	52.8 ± 26.2	90.2 ± 1.4

Coefficient of variation = 25.38%.

LSD-10.461 (0.05), 13.748 (0.01); p≤0.05.

**Table 2:** **IC**_**50**_**(μg/ ml) values for VLC fractions of*C. globosum*EF18 tested against*S. sclerotiorum***

Sl no.	Extract fraction	4^th^ day			5^th^ day		
		IC_50_	χ^2^	Regression equation	IC_50_	χ^2^	Regression equation
1	WSL 2E IV	135.43	0.115	Y = 2.711 + 1.272 X	260.31	0.577	Y = 4.818 + 1.995 X
2	WSL 2E V	114.71	1.189	Y = 2.132 + 1.035 X	281.95	0.841	Y = 5.803 + 2.368 X
3	WSL 2E VI	158.74	2.037	Y = 3.132 + 1.423 X	243.86	5.635	Y = 5.895 + 2.469 X
4	WSL 2E VII	166.84	0.001	Y = 4.292 + 1.931 X	209.56	14.76	Y = 7.104 + 3.061 X
5	WSL 2E VIII	40.57	1.165	Y = 1.97 + 1.225 X	35.4	0.696	Y = 2.228 + 1.438 X
6	WSL 2E IX	155.26	0.399	Y = 3.008 + 1.373 X	80.14	0.333	Y = 2.351 + 1.235 X
7	WSL 2E X	220.44	1.301	Y = 6.302 + 2.689 X	179.31	0.414	Y = 6.307 + 2.799 X

**Figure 3:**
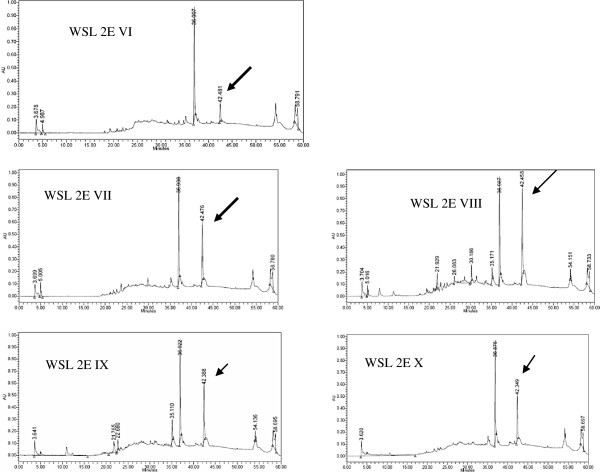
**HPLC chromatogram of VLC fraction of ethyl acetate extract of*C. globosum*EF18, obtained with Waters HPLC and showing the peak of compound (Rt 42.3) responsible for antifungal activity.**

**Figure 4:**
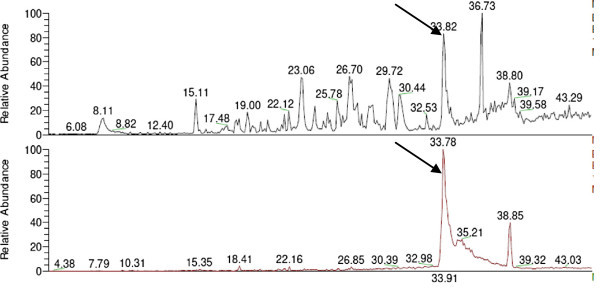
**LC-MS chromatogram of purified fraction (WSL 2E VI_I_S1) obtained from preparative HPLC.**

**Figure 5:**
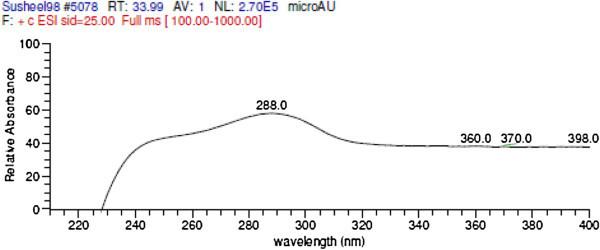
**UV spectrum of the compound ‘A’.**

**Figure 6:**
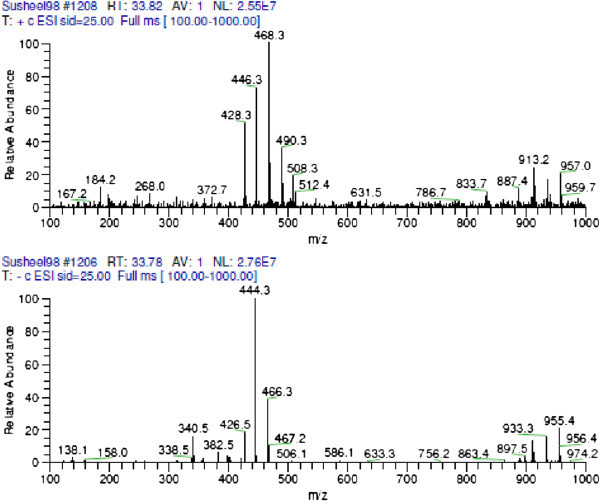
**m/z (H**^**+**^**) spectra of the purified compound ‘A’.**

**Figure 7:**
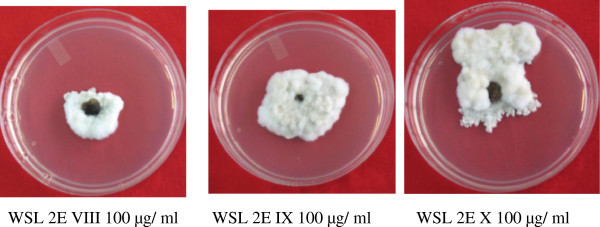
**Effect of different VLC fractions obtained from ethyl acetate extract of*C. globosum*EF18 on radial growth of*S. sclerotiorum.***

The VLC fractions VI- X showing activity against *S. sclerotiorum* were having a common peak at RT 42.3-42.4 minutes (from Waters HPLC) (Figure 3). Correlation of chemical analysis and bioassay results, it is apparent that the compound eluting at RT 42.5 is responsible for the antifungal activity. As depicted in the Table 3, increased concentration― corresponds to increased area of the peak― of this compound in the fraction was prime cause of increased bioactivity of the fraction. Table 4 shows the regression model and correlation between increasing concentration of active compound in the fraction and bioactivity of fraction. Correlation coefficient (r) of 0.86 was calculated between the % area of the compound in the HPLC chromatogram and biological activity at 100 μg/ml, which says that statistically 86% of the activity in these fractions is due to the compound and rest 14% is due to the unexplained reason. LC-MS analysis suggested the molecular weight of the compound associated with peak (RT 33.78 in LC-MS) as 445 (m/z^+^ 446 and m/z^-^ 444) and UV maxima 226 and 288 nm (as presented in Figures 4, 5 and 6). Figure 7 shows the plate photograph of growth inhibition in *Sclerotinia sclerotiorum* due to the toxicant present in VLC fractions VIII, IX and X.

**Table 3:** **Effect of increasing concentration of antibiotic sch210971 on GI property of VLC fractions against*S. sclerotiorum***

Fraction no.	% Peak area at^*^Rt 42.4 min (λ_max_ 288 nm)	Growth inhibition (%)		
		100 μg/ml	200 μg/ml	500 μg/ml
WSL 2E IV	-	0.0	0.0	47.84
WSL 2E V	-	0.0	0.0	33.72
WSL 2E VI	8.88	0.0	3.92	74.90
WSL 2E VII	27.6	0.0	44.31	75.29
WSL 2E VIII	30.34	76.4	82.63	88.80
WSL 2E IX	20.00	55.19	64.70	84.87
WSL 2E X	18.48	21.57	55.74	86.55

*This Rt (from Waters HPLC, C-18) corresponds to Rt 34.1 (from Dionex HPLC) of above chromatogram.

**Table 4:** **Correlation coefficient and regression model between % peak area of sch210971 in different VLC fractions and their growth inhibition property against*S. sclerotiorum***

Fraction no.	% Peak area at Rt 42.4 min (λ_max_ 288 nm)	% Growth inhibition at 100 μg/ml	% Growth inhibition at 200 μg/ml
WSL 2E VIII	30.34	76.4	82.63
WSL 2E IX	20.00	55.19	64.70
WSL 2E X	18.48	21.57	55.74
Regression Model		% GI= −33.469 + 3.684 X % Peak area ± 19.952	% GI = 20.194 + 2.070 X % Peak area ± 4.14
Coefficient of determination (R)		0.740	0.954
Coefficient of correlation (r)		0.860	0.976

**Figure 8:**
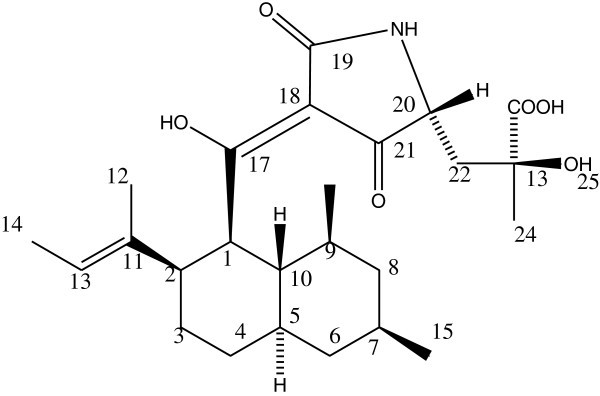
**Structure of the antibiotic Sch 210971.**

WSL-2E_VI _I_S1 fraction was subjected to preparative HPLC to obtain pure compound ‘A’, which was further analyzed by NMR spectroscopy. Comparative analysis of NMR and MASS revealed that compound ‘A’ is similar to Antibiotic Sch 210971 (m/z 445 and λ_max_ 290) (Figure 8), which has previously been isolated from *C. globosum* by Yang et al. (2006). In the finding by Yang et al. molecular weight of the isolated compound was 445 Da and showed the protonated molecular ion at m/z^+^ 446 similar to the compound isolated by us. Also UV absorption of Sch 210971 at 220 and 295 was near to that of our compound i.e. 226 and 288.

## Discussion

Hexane extract of *C. globosum* has been reported as antifungal against *S. sclerotiorum* and *Botrytis cineria* (Nakashina et al. 1991). However we find polar extract viz. methanol and ethyl acetate were more active than hexane extract against *S. sclerotiorum*. Antibiotic Sch 210971 has been previously reported as chemokine receptor CCR-5 inhibitor (Yang et al. 2006); however this is the first report of antifungal activity of this compound. HPLC has been utilized for the first time in our study in finding out the principle component of *C. globosum* responsible for antifungal activity. Previously chaetomugilin D, together with three known metabolites, chaetomugilin A, chaetoglobosins A and C have been isolated by a bioassay-guided fractionation from the EtOAc extract of the cultures of *C. globosum*. Chaetomugilin D also reported to have antifungal activity against *Mucor miehei* (Qin et al. 2009). *C. globosum* an endophyte to a medicinal plant *Curcuma wenyujin* has yielded chaetoglobosin X, which showed broad antifungal activity (Wang et al. 2012). Zhang et al. (2013) have also isolated chaetoglobosins A and C from *C. globosum* and they found that chaetoglobosin A is having antifungal activity against *Setosphaeria turcica*, causal agent of northern corn leaf blight. Two other antifungal substances viz. chaetoviridins A and B have been purified from culture broth of *C. globosum* isolated from barnyard grass. Chaetoviridin A exhibited higher antifungal activity with 80% reduction in disease development at 62.5 μg/ml concentration against rice blast and wheat leaf rust and 50% control of late blight of tomato at 125 μg/ml (Park et al. 2005), however purified fraction isolated in our experiment has IC_50_ value of 35.4 μg/ml. In present study we have found that Antibiotic Sch 210971 can be used as marker for antifungal activity against *S. sclerotiorum*.

Role of *C. globosum* in biological control has been well documented and commercial formulation has also been developed (Soytong et al. 2001). Culture filtrate of *C. globosum* has been reported to successfully inhibit the mycelial growth of *Pythium ultimum* in In vitro and pot culture experiments. Chaetomin has been the principle compound responsible for this antifungal activity (Di-Pietro et al. 1992). Cell wall degradation caused by beta-glucanases and carboxymethyl cellulases is one of the possible modes of action of *C. globosum* against *P. ultimum* (Inglis and Kawchuk 2002). Mode of action of Antibiotic Sch 210971 needs to be studied further. Antibiotic Sch 210971 enriched fraction can be formulated as antifungal biopesticide.

## Materials and methods

HPLC was performed with Chromeleon Ver 6.3 program; Dionex P580A LPG Pump; Photo Diode Array (PDA) Detector UVD 340S detector; ASI-100T Autosampler; STH 585 Column Thermostat; Eurospher 100-C18, Knauer column. HPLC of extracts was also performed on Waters HPLC system with Autosampler, 717 plus; PDA 2996 detector; System controller 600 and Empower2 software with polar gradient method. The samples were analyzed on a Phenomenex column (250 × 4.60 mm, 5 μ) using mobile phase acetonitrile: water (HPLC grade). Preparative HPLC was done with Varian prepstar 218 pump; Microsorb 60–8 C18 column with Varian Prepstar 320 detector and Rheodyne 7725i injection block. LC-MS was performed on Agilent 1100 series HPLC system (pump, detector and autosampler) with Knauer (125 mm L, 2 mm ID), prepacked with Eurosphere- 100 C-18 (5 μm) and with integrated pre-column and Finnigan LC Q-DECA MS detector. HPLC grade methanol and nanopure water with ortho-phosphoric acid 0.15%, pH 2.0 were used for HPLC and LC-MS. ^1^H NMR was recorded on Bruker DRX-500 instrument operating at 500 MHz.

### Batch culture fermentation of *C. globosum* EF18 and its extraction

**Figure 9:**
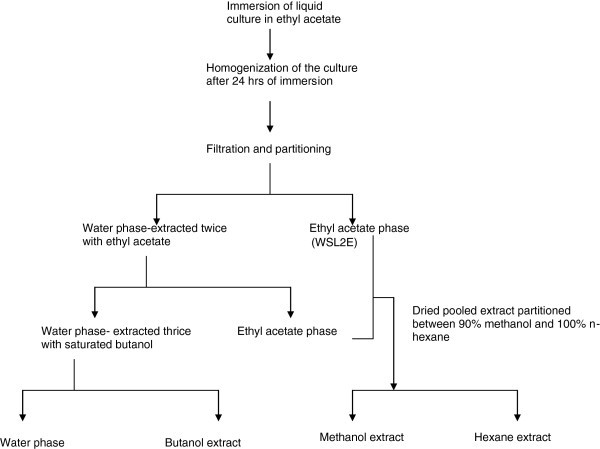
**Schematic diagram of extraction procedure for obtaining crude fungal extracts of*C. globosum*EF18 (WSL2).**

Fungus was multiplied in 11.7 litres of wickerham medium [Malt extract (3 g/l); Yeast extract (3 g/l); Peptone (5 g/l); Glucose (Qualigens)-10 g/l; pH-7.2-7.4] at 24°C for 3–4 weeks. Media chemicals were purchased from Himedia, India. One flask of medium without the inoculum was kept as control. Metabolite extraction was done as per the protocol of Wicklow et al. (1998). Extraction procedure has been depicted in Figure 9 as flow diagram.

### Isolation of antifungal compound

**Figure 10:**
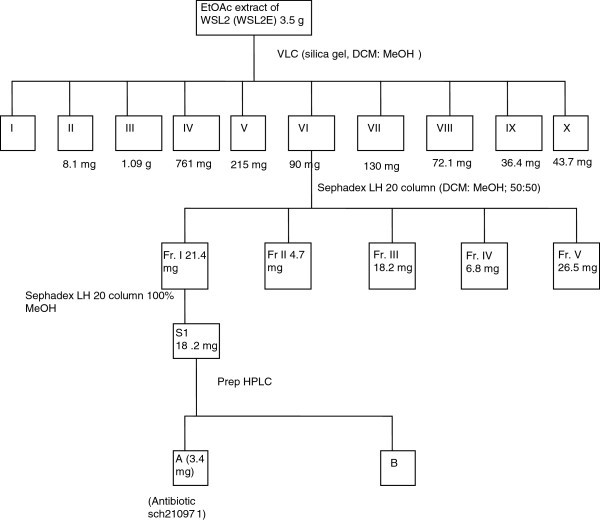
**Schematic diagram of procedure of separation/ purification of ethyl acetate extracts of*C. globosum*isolate EF18 (WSL2E).**

A schematic diagram of procedure for separation and purification is presented in Figure 10. Extract was subjected to VLC and eluted with dichloromethane: methanol in the following order: 100% DCM, 95:5 DCM: MeOH, 90:10, 80:20, 70:30, 60:40, 50:50, 30:50, 25:75 and 100% MeOH. These fractions (WSL 2E I-X; Figure 10) were dried in rotary under vacuum and investigated by TLC and HPLC. Based on the HPLC and LC-MS profiles of the fractions, fraction no. WSL2E VI was further purified by sephadex LH 20 (Merck) column with dichloromethane and methanol 50:50. Based of TLC pattern, fractions were grouped in to 5 groups- WSL2EVI_I-V. These 5 fractions were analyzed by HPLC and LC-MS. Sub-fraction 1 (WSL2EVI_I) was further purified by sephadex column with 100% methanol followed by preparative HPLC (Figure 10). Preparative HPLC was done with the following gradient 0–5 min 50% methanol and 5–35 min increase from 50–100% of methanol and from 35–40 100% methanol. One major compound was obtained (compound ‘A’ in Figure 10).

### Identification of antifungal Sch 210971

The Identification was done by MASS and NMR. The sample was dissolved in methanol and injected to HPLC/ESI-MS hyphenated system. This compound corresponds to compound of Rt. 42.5 from Waters HPLC, C-18 and to Rt 34.1 from Dionex HPLC. The compound eluted at Rt 33.91 having m/z^+^ 446.3, λ_max_ 288 nm.

NMR measurement carried out at Heinrich Heine Universitat, Dusseldorf, Germany. Deuterated methanol was used to dissolve samples for NMR measurement. 1D and 2D NMR spectra were processed and analyzed using NMR software 1D WIN-NMR and 2D WIN-NMR Bruker NMR suite. NMR spectra were calibrated using solvent signals of their protons. Observed chemical shifts value (δ) were given in ppm and coupling constant *J* in hertz (Hz).

^1^H NMR of the compound ‘A’ was generated with methanol-d at 500 MHz and found as δ 5.84 (1H, S), 5.75 (1H, s), 5.59 (1H, s), 5.48 (s), 5.24 (s), 4.38 (1H, d,), 4.29 (1H, dt, J= 7.6, 6.3), 3.81 (dd), 3.62 (1H, d), 2.9 (1H, br s), 2.65 (1H, s), 2.34 (1H, d, J=12), 2.19 (s), 2.13 (1H, d, J= 12.5), 1.92 (3H, d, J= 9.8), 1.74 (3H, br s), 1.61 (5H, s), 1.57 (s), 1.47 (6H, d, J= 10.15), 1.4 (2H, d, J= 12.2), 1.28 (s), 1.23 (s), 1.18 (s), 0.89 (5H, d, J= 2.8), 0.79 (3H, d, J=4.2). Comparative ^1^H NMR of the purified compound and published compound is provided in Table 5.

**Table 5:** **Comparison of**^**1**^**H NMR of purified fraction and compound ‘A’ from literature (Yang et al.**^2006^**)**

Atom no.	Chemical shift H’		Multiplicity		Coupling constant	
	Compound ‘A’	Literature	Compound ‘A’	Literature	Compound ‘A’	Literature
8b		0.83		M, 1H		
16	0.79	0.85	D 3H	D, 3H	4.2	6.5
15	0.89	0.91	D 5H	D, 3H	2.8	6.5
6b		0.95		M, 1H		
	1.18		S			
	1.23		S			
	1.28		S			
9		1.39		M, 1H		
10	1.4	1.4	D 2H	M, 1H	12.2	
24	1.47	1.49	D 6H	S, 3H	10.15	
14		1.5		Br d, 3H		6.5
12	1.57	1.57	S	Br s, 3H		
7		1.63		M, 1H		
8a	1.61	1.66	S 5H	M, 1H		
22b	1.74	1.75	BR S 3H	Dd, 2H		
5		1.85		M, 1H		
6a	1.92	1.91	D 3H	M, 1H	9.8	
	2.13		D 1H		12.5	
	2.19		S			
22a	2.34	2.5	D 1H	Dd, 1H	12.0	14.0, 2.5
	2.65		S 1H			
2	2.9	3.00	BR S 1H	Dt, 1H		8.0, 1.0, 1.0
	3.62		D 1H		12.45	
20	3.81	3.8	DD	Dd, 1H		10.0, 2.5
1	4.29	3.94	DT 1H	Dd, 1H	7.6, 6.3	8.0, 7.0
	4.38		D 1H			
13	5.24	5.19	S	Dq, 1H		6.5, 1.0
	5.48		S			
3	5.59	5.66	S 1H	Br s, 2H		
4	5.75		S 1H			
	5.84		S 1H			

### Bioassay of extracts/fractions of *C. globosum* EF18 against *Sclerotinia sclerotiorum*

Bioassay of crude extract was done as per the procedure described by Kumar and Kaushik (2013). Whereas, for bioassay of fractions 30 mg of dried extract was dissolved in 600 μl of methanol and out of this 60, 120 and 300 μl were mixed to 30 ml of media for 100, 200 and 500 μg/ml concentrations. There were 3 replications for each treatment.

### Data analysis

Percent growth inhibition (GI) and analysis of variance of the GI was performed as described earlier (Kumar and Kaushik 2013). Concentration at which 50% growth inhibition occurs i.e. IC_50_ was calculated by probit analysis (Finney 1971) using the software SPSS 17.0.
